# Editorial: Insights in movement science and sport psychology 2021

**DOI:** 10.3389/fpsyg.2023.1267376

**Published:** 2023-09-05

**Authors:** Guy Cheron, Sergio Machado, Maurizio Bertollo

**Affiliations:** ^1^Laboratory of Neurophysiology and Movement Biomechanics, Université Libre de Bruxelles, Brussels, Belgium; ^2^Laboratory of Physical Activity Neuroscience, Neurodiversity Institute, Federal University of Santa Maria, Santa Maria, Brazil; ^3^Behavioral Imaging and Neural Dynamics Center, G. d'Annunzio University of Chieti–Pescara, Chieti, Italy

**Keywords:** sports, ecological, dynamic system, creativity, motor action, emotion, performance

The purpose of this Research Topic is to highlight recent research developments in the field of movement science and sports psychology. Even though they are all centered on the practice of sport, the various studies published in this Research Topic demonstrate the great diversity of the themes addressed and the scientific tools used to better understand the foundations of sports activity and its various implications on human health and society in general.

The convergence of ecological, cognitive, and dynamic systems theories to movement science promotes the study of creativity in sports (Zahno and van der Kamp). The old debate of the “motor-action controversy” is revisited to reach a conceptual proposal in which optimal feedback and feedforward predictive controls meet the dynamic systems theory of ecological psychologists, integrating the person-environment relationship.

Basic neurophysiology is here developed around the effects of hypoxia on cognitive performance (Guicciardi et al.) demonstrating that hypoxia increased reaction times, but has no effects on mood state.

Decision-making occupies a central position in the perceptual-cognitive factors and is considered a predictor of talent. This aspect is studied (Hinz et al., 2022) in which experts and non-experts handball players were asked to respond as soon as possible to different attack sequences. This study demonstrates that the experts responded significantly more often with offensive responses than the non-experts. However, the level of expertise does not affect the decision time which decreases with increasing visual information. Six key points (analytical decisions, visual search strategies, creativity, emotions, development, and team coordination) of intuitive decision-making are proposed as determinants of expertise in athletes, referees, and coaches in naturalistic sports (Bossard et al.).

It is now well-recognized that physical exercise (PE) improves not only physical health but also cognitive function, the risk reduction of neurological diseases, and the detrimental effects of aging. In addition, PE improves verbal and graphic fluency and enhances emotional awareness, self-efficacy and self-esteem, the ability to express emotion, and the enhancement of stress management (Passarello et al.).

The effects of real and imagined endurance exercises on sustained attention performance (Wieland et al.) demonstrated that the combination of these two different physiological states (endurance exercise and motor imagery) contribute to better cognitive performance.

The study of the mental representation of overhead throwing movement (Gromeier et al.) demonstrates that the building blocks of mental representations including functional, sensory, spatiotemporal, and biomechanical characteristics of a movement are acquired progressively with age and practice.

Sports injuries and rehabilitation occupy an important sector of human health integrating a large number of medical, individual identity, sport specificity, demographic, and psycho-social factors. In this context, a large cohort of athletes suffering from anterior cruciate ligament injury (McGinley et al.) are studied. It is shown that the Athletic Identity Measurement Scale (AIMS) significantly depends on the sex, years active in sport, activity level, and ACSI-Coachability. Along the same line, a case study (Gomez-Espejo et al.) illustrates the multifactorial aspects of sports injury concerning the emotional and psychological treatment of pain management. Despite the tentative to standardize the methodology for the recovery process following injury and the rapid return to competition, new strategies integrating interdisciplinary approaches are presented (Brooks et al.).

The study of the functional links between working memory and biological motion (Wang et al.) demonstrates that the working memory capacity depends on a large number of factors including the presentation duration, the number of joints, the limb, and the articulations used.

The attractiveness of some sports practices such as the marathon and related touristic activities opens a new field of psychosocial studies of the complex relationships between recreation specialization, life satisfaction, psychological commitment, and social support (Tian et al.).

New recommendations about sudden and unexpected significant declines of performance in the field of “choking under pressure” for individual and team situations are given (Wergin et al.).

The time pressure exerted on adolescent athletes merits to be taken into account in the occurrence of burnout and it is proposed that leisure activities are excellent countermeasures to ensure better functional and mental development and brain maturation in adolescents (Vacher et al.).

## Author contributions

GC: Writing—original draft. SM: Writing—review and editing. MB: Writing—review and editing.
